# Solid-phase synthesis of molecularly imprinted polymer nanolabels: Affinity tools for cellular bioimaging of glycans

**DOI:** 10.1038/s41598-019-40348-5

**Published:** 2019-03-08

**Authors:** Paulina X. Medina Rangel, Sylvain Laclef, Jingjing Xu, Maria Panagiotopoulou, José Kovensky, Bernadette Tse Sum Bui, Karsten Haupt

**Affiliations:** 1 Sorbonne Universités, Université de Technologie de Compiègne, CNRS Enzyme and Cell Engineering Laboratory, Rue Roger Couttolenc, CS 60319, 60203 Cedex Compiègne, France; 2 Université de Picardie Jules Verne, Laboratoire de Glycochimie, des Antimicrobiens et des Agroressources, UMR CNRS 7378, 33 rue Saint Leu, 80039 cedex Amiens, France

## Abstract

Hyaluronic acid (HA) is a glycosaminoglycan that plays many roles in health and disease and is a key biomarker of certain cancers. Therefore, its detection at an early stage, by histochemical methods, is of importance. However, intracellular HA can be masked by other HA-binding macromolecules, rendering its visualization somehow problematic. We show that fluorescent molecularly imprinted polymer nanogels (MIP-NPs), can localize and detect intracellular HA. MIP-NPs were synthesized by solid-phase synthesis on glass beads (GBs). GBs were functionalized with terminal alkyne groups on which an azide derivative of the template molecule glucuronic acid was immobilized via click chemistry. Immobilization via the anomeric carbon left the template’s carboxyl moiety free to enable strong stoichiometric electrostatic interactions with a benzamidine-based functional monomer, to confer selective recognition to the MIP-NPs. Due to the two-point orientation of the template, the resulting MIP-NPs were endowed with improved binding site homogeneity and specificity, reminiscent of monoclonal antibodies. These synthetic antibodies were then applied for probing and staining HA, of which glucuronic acid is a substructure (epitope), on human epidermal cells. Their excellent sensitivity, small size and water compatibility, enabled the MIP-NPs to visualize HA, as evidenced by confocal fluorescence micrographs.

## Introduction

Molecularly imprinted polymers (MIPs) are tailor-made antibody mimics obtained by a templating process at the molecular level^1–3^. They are synthesized by copolymerizing functional and cross-linking monomers around a template molecule. This leads to a 3D polymer network containing stable cavities that are complementary to the template in terms of size, shape, and position of functional groups. Thus a “molecular memory” is introduced into the polymer, allowing the molecular recognition and binding of target analytes with a high affinity and specificity. MIPs have gained popularity ever since Mosbach’s group, in 1993, reported their application in a pseudo-immunoassay for the determination of drugs^4^, whereby for the first time, MIPs were coined ‘antibody mimics’^5^. Hence, MIPs have been around for some time now and they have been widely developed and applied in solid-phase extraction^6,7^, sensors^8,9^, pseudo-immunoassays^10,11^, drug delivery^12,13^ and very recently for optical bioimaging^14–16^. Nevertheless, despite many efforts to make MIPs become ‘*a useful general alternative to antibodies*’^5^, they are still not very much commercialized. The main drawbacks is their non-compatibility with water, their incomplete template removal and the non-homogeneity of their binding sites.

To overcome these problems, the recently-developed solid-phase synthesis approach in which the template is covalently immobilized on glass beads (GBs) as solid support, has emerged as a promising solution^17,18^. This configuration can allow an oriented immobilization of the template upon which MIP-NPs are synthesized^19–21^. The GBs play the role of both a reactor and a separation column since the MIP-NPs are synthesized and purified *in situ*. After synthesis and removal of unreacted reagents and nanoparticles that polymerized distantly from the immobilized template, uniform MIP-NPs with homogeneously oriented binding sites are eluted. The MIP-NPs are soluble in water if hydrophilic precursors are used, and more so if the polymerization is carried out in an aqueous mixture. These nanoMIPs can thus be considered analogous to monoclonal antibodies, endowed with a high affinity (K_D_ ~ pM-nM)^20^.

In this work, we focus on the fluorescence-based bioimaging of hyaluronic acid (HA), in human keratinocytes (HaCaT cell line). Hyaluronic acid (HA) is the predominant glycosaminoglycan (GAG) in human skin^22^ and participates in lubricating joints or holding together gel-like connective tissues^23^. It also co-regulates cell behavior during healing processes, inflammation, tumor development, and several reports have highlighted HA as the key biomarker of certain cancers^23,24^. Therefore, the possibility to localize, detect and quantify HA at an early stage would be useful for diagnostics and therapy. However, HA is not immunogenic, thus raising antibodies against it is problematic. Typically, a specific probe for HA, called hyaluronic acid binding protein (HABP) is used for histochemistry^25,26^. Staining is done in two steps, first with a biotinylated HABP, followed by incubation with streptavidin-FITC. Besides being rather expensive, these biological probes have a short shelf-life and must be conserved at −20 °C. In this context, we propose to use rhodamine-labeled MIP-NPs which will stain HA, in a single step. Besides, the MIP-NPs possess other advantages as they are physically and chemically stable, are cheap to fabricate and their sizes can be tuned for targeting either extracellular or intracellular HA. We used D-glucuronic acid (GlcA), a substructure (epitope) of HA (Fig. S1), as the imprinting template. GlcA is also present in proteoglycans such as chondroitin sulfate and heparan sulfate, but in human keratinocytes, the majority is found in HA. Indeed, in healthy human skin, HA represents 65% of the total content of GAGs, whereas chondroitin sulfate represents only 5%. The second most abundant GAG in human skin is dermatan sulfate, which represents 22% but does not contain GlcA^27^.

**Figure 1:**
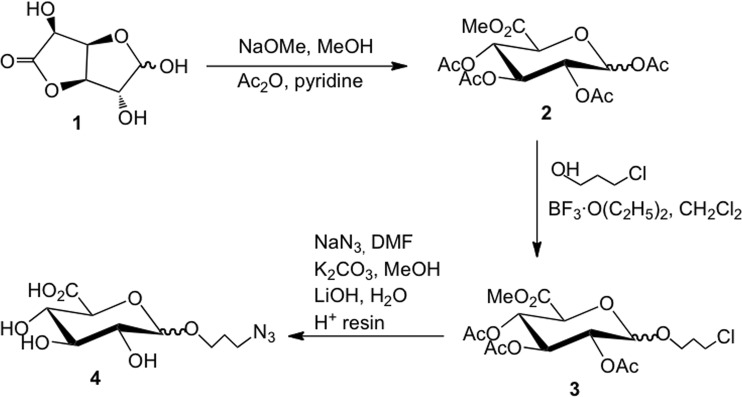
Synthesis of azidopropyl glucuronic acid **4** from commercial precursor molecule **1**.

**Figure 2:**
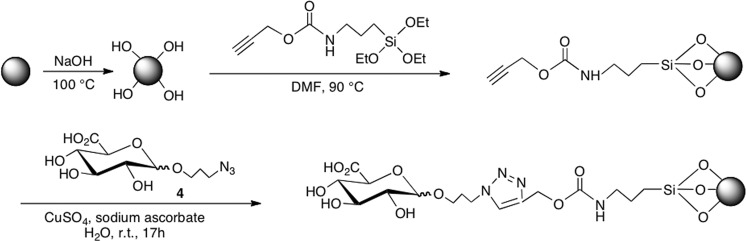
Immobilization of azidopropyl glucuronic acid **4** on propargylated GBs *via* copper (I) catalyzed Huisgen 1,3-dipolar cycloaddition (CuAAC) reaction (click chemistry).

Undoubtedly, solid-phase synthesis will be widely adopted for producing MIPs in the future. However, only a few linking chemistries^18^ for immobilizing the template on the solid support has been employed so far. Herein we report that click chemistry can constitute an interesting alternative to immobilize the template on GBs. First, GlcA bearing an azide moiety on its anomeric carbon was synthesized (Fig. 1), to enable its subsequent immobilization on propargylated-GBs *via* click reaction (Fig. 2). In order to confer high selectivity to the MIP, a functional monomer bearing a benzamidine moiety, (4-acrylamidophenyl) (amino)methaniminium acetate (AB), which forms very strong electrostatic interactions in a 1:1 stoichiometry with the –COOH moiety of the template^15^ (Fig. S2), was included in the polymerization mixture. A rhodamine fluorescent monomer and a high amount of *N*-isopropylacrylamide (NIPAM) was added to the MIP mixture to respectively impart fluorescence and thermoresponsiveness, allowing the facile liberation of the MIP-NPs from the immobilized template by a simple temperature change. The MIP was very specific toward GlcA as negligible binding was observed with the non-imprinted polymer (NIP). MIP staining of keratinocytes was confirmed by treatment with hyaluronidase which degrades hyaluronan. Subsequent staining with MIP-NPs or the HABP probe, coupled with steptavidin-FITC, yielded similar results.

## Results and Discussion

### Synthesis of azidopropyl glucuronic acid

The template, azidopropyl glucuronic acid **4** (Figs 1, S3 and S4) was obtained in three steps, according to a previously described procedure^28^, modified to proceed without the purification of intermediates.

### Preparation of the column reactor

The solid-phase synthesis approach is represented in Fig. 2. GBs were first activated by boiling in NaOH to introduce –OH groups^20,21^. After activation, they were functionalized with O-(propargyloxy)-N-(triethoxysilylpropyl)urethane so as to introduce terminal alkyne groups^29^, for subsequent immobilization of azidopropyl glucuronic acid by click chemistry via CuAAC reaction, yielding a stable 1,2,3-triazole. CuAAC reaction is fast, regioselective, simple to apply, and gives high product yields^30,31^. Since imprinting small molecules by the solid phase approach requires that the template be immobilized on the solid support without involving the functional groups that can be exploited for recognition properties, an alkyne or azide equivalent can be a useful alternative for its immobilization on solid support without compromising the accessibility of the functional groups for the imprinting process. For sugars, the anomeric carbon can be conveniently used to attach the coupling group. This strategy is herein employed for the first time to immobilize a template molecule for the obtention of MIP-NPs using solid-phase synthesis. Previously, templates bearing an –NH_2_ or –COOH or –SH groups were immobilized by forming a Schiff base with glutaraldehyde or *via* ethylcarbodiimide/N-hydroxysuccinimide (EDC/NHS) or succinimidyl iodoacetate coupling^18^, respectively.

To verify that free alkyne groups have been grafted on the GBs, click chemistry in the presence of CuSO_4_/sodium ascorbate, was performed with a fluorescent azide dye, coumarin 343 azide (Fig. S5). The amount of clicked fluorescent azide was determined in the supernatant containing the non-reacted dye and found to be 97.8 ± 3.3 nmol (n = 4) per g of GBs. Activated GBs without any functionalization served as blanks.

### Solid-phase synthesis of MIP-NPs

Having ‘clicked’ azidopropyl glucuronic acid to alkyne GBs, the resulting GlcA-GBs were packed in a column with a thermostated jacket, equipped with two adapters for regulation of the bed volume. After equilibrating the column with 25 mM sodium phosphate buffer, pH 7.0 (buffer A), the stoichiometric monomer AB was pumped through the column at a slow rate to favor template-monomer interaction. AB forms strong 1:1 electrostatic interaction with the -COOH moiety of glucuronic acid, with an association constant K_a_ of 7.1 × 10^3^ M^−1^ ^15^. The rest of the polymerization mixture, composed of the functional monomer NIPAM, the fluorescent monomer rhodamine B, the cross-linker EbAm and the initiator system (KPS, TEMED) in buffer A, was then passed through the column. NIPAM is a functional monomer capable of hydrogen bond interactions, due to the presence of oxygen and nitrogen atoms^32,33^, but at the same time was used herein as the major component in this polymer recipe to obtain thermoresponsive MIP-NPs. The lower critical solution temperature of the polymer was determined by dynamic light scattering by measuring the particles size versus temperature and found to be ∼32 °C (Fig. S6). The imprinted polymer was synthesized at 37 °C, and the growing polymeric nanoparticles, which are in the collapsed state at this temperature, encapsulate the immobilized glucuronic acid during polymerization. After polymerization, the column reactor was washed with buffer A at 37 °C to remove unreacted reagents and particles that polymerized distantly from the immobilized template. The reactor was then washed with the same buffer at room temperature (25 °C), allowing the MIP-NPs to swell and be eluted. The yield of polymerization of the MIP-NPs was 0.2 ± 0.03 mg per g of GBs (n = 3). Control NIP-NPs were synthesized following the same protocol as described above but in the absence of glucuronic acid.

### Physicochemical characterization of MIP-NPs

**Figure 3:**
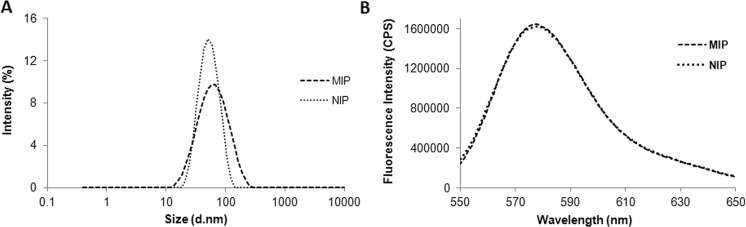
(**A**) Size distribution as measured by dynamic light scattering in 25 mM sodium phosphate buffer pH 7.0 and (**B**) fluorescence emission spectra (λ_ex_ = 540 nm), of MIP-NPs and NIP-NPs in water.

MIP-NPs were obtained as a transparent solution. No aggregation or change in fluorescence was observed for a period of six months when stored at 4 °C in the dark. The hydrodynamic size of the MIP-NPs was analyzed by dynamic light scattering (DLS). The diameters of MIP-NPs and NIP-NPs were 70.69 ± 4.7 nm and 55.56 ± 2.9 nm respectively (Fig. 3A).

The fluorescence properties of MIP and NIP were found to be similar (Fig. 3B). A fluorescent dye moiety was incorporated into the polymer matrix by adding a polymerizable rhodamine derivative to the polymerization mixture. Its molar ratio with respect to the other monomers has been previously optimized to maximize the fluorescence intensity of the particles (optimal ratio 1:0.05, AB:rhodamine); higher dye content resulted in lower brightness due to reabsorption or energy transfer^34^.

### Evaluation of the binding properties of MIP-NPs

**Figure 4:**
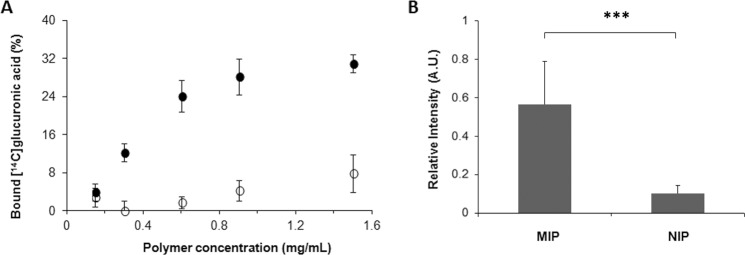
(**A**) Equilibrium binding isotherms in water of [^14^C]D-glucuronic acid to MIP-NP_S_ (full circles) and NIP-NPs (empty circles) at 37 °C. Data are means of two independent experiments, with two repetitions for each point, for two batches of polymers (n = 4), error bars present standard deviations. (**B**) Relative fluorescence intensity of keratinocytes after imaging with MIP-NPs and NIP-NPs (n = 3 independent replicates for each experiment). Mean values of MIP and NIP are significantly different at 95% confidence (***p < 0.001, Student’s t-test with unequal variance).

The recognition properties of MIP-NPs were evaluated by equilibrium radioligand binding assays with [^14^C]D-glucuronic acid in water. Figure 4A shows that MIP-NPs bound specifically to GlcA, as negligible binding was observed with NIP-NPs, thus indicating the creation of imprinted sites. Non-linear fitting of the data to a single-site Langmuir binding isotherm yielded a dissociation constant (K_D_) of ∼800 nM, after determining the molecular weight of the MIP by the Debye plot, to be 625 ± 35 kDa (Fig. S7). Thus MIP-NPs obtained by oriented solid-phase imprinting advantageously present a lower K_D_ than MIPs prepared by precipitation polymerization (AB, methacrylamide, ethylene glycol dimethacrylate, DMSO), with a K_D_ of 196 µM^34^.

Previous competitive binding experiments with glucose, galactose, N-acetylglucosamine, N-acetylgalactosamine and N-acetylneuraminic acid (sialic acid), other monosaccharides that are present on the cell surface, indicated that MIPGlcA was very selective towards GlcA as only <1% cross-reactivity was observed^15,34^. Therefore, the MIPGlcA was confidently applied for selective bioimaging of HA.

### MIP-NPs for imaging fixed cells

**Figure 5:**
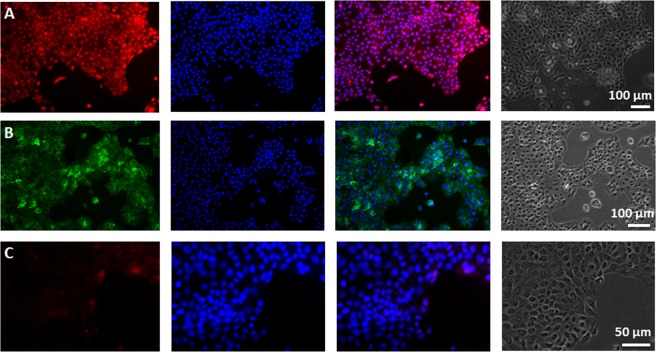
Representative epifluorescence microscope images of confluent HaCaT cells that were fixed and stained with (**A**) MIP-NPs, (**B**) FITC-labeled hyaluronic acid binding protein (HABP) and (**C**) NIP-NPs. From left to right: staining by rhodamine-labeled polymers (red) or streptavidin FITC-labeled HABP (green), cell nucleus counterstained with Hoechst (blue), the corresponding merge image and the phase contrast.

A standard immunostaining protocol^15,34^ was adopted for the application of MIP-NPs for cell imaging, to localize HA on HaCaT cells. The fixation of cells is based on paraformaldehyde, which has a low background fluorescence. A blocking step is then performed with glycine, to stop the fixation and reduce nonspecific binding. The final step is the incubation with MIP-NPs, or with NIP-NPs as a control. The spatial distribution and localization of the particles was determined by epifluorescence and confocal microscopies. Figure 5 shows that the particles were located exclusively in regions where cells were present. To compare with a reference method for HA localization on the cells, a biotinylated hyaluronic acid binding protein was used. The protein was revealed with FITC-labeled streptavidin. We can clearly see that MIP staining (red) (Fig. 5A) compares very favorably with that of HABP/streptavidin-FITC (green) (Fig. 5B). On the other hand, NIP-NPs which contain no recognition sites for GlcA do not stain the cells (Fig. 5C), following the prediction of Fig. 4A.

**Figure 6:**
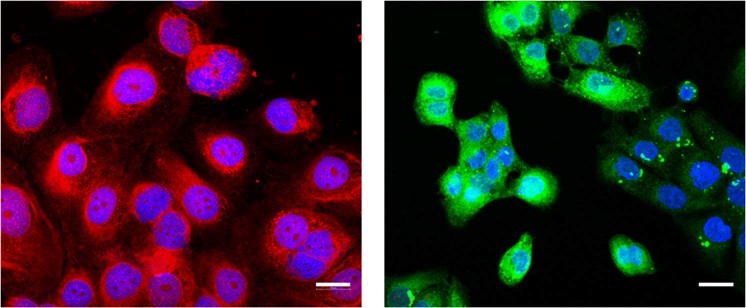
Confocal images of fixed human keratinocytes showing extracellular, intracellular and nuclear labeling by MIPGlcA-NPs (red) (left) and by HABP/streptavidin FITC (green) (right), nuclear counterstaining with Hoechst (blue), scale bar: 20 µm.

Fluorescent particles were quantified by epifluorescence microscopy on individual cells (see *Methods* for details). A background subtraction was applied in order to determine the fluorescence signal coming from the particles. As a result, MIP-NPs showed six times more binding to the cells than NIP-NPs (Fig. 4B). Confocal microscopy was then used to study the distribution of MIP-NPs along the z axis. As shown in Fig. 6, the nanoparticles were mainly localized pericellularly and intracellularly, even within the nucleus. Nuclear staining, due to the distribution of hyaluronic acid in nuclear clefts, has been reported previously^25^. Intracellular HA plays an important role in the intracellular and nuclear structure during mitotic stages, along with inflammatory processes^35^. However, its localization inside the cell remains challenging because of its lack of accessibility due to masking by extracellular proteoglycans and glycoproteins (HA-binding molecules). Thus, our MIP, due to its small size of ∼70 nm, can advantageously access and probe intracellular HA. Though HA is the predominant GAGs in human skin^22^, other GAGs containing GlcA like chondroitin sulfate and heparan sulfate, may have also been stained by the MIP-NPs, but to a low extent as they represent only 5% of total GAGs as compared to 65% of HA. In fact, dermatan sulfate, the second most abundant GAG in human skin^27^, consists mostly of repeating units of iduronic acid and galactosamine, whereas chondroitin sulfate is found in smaller amounts compared to HA and dermatan sulfate^36^. Evidence of HA staining was further strenghtened by the reduction of the staining with MIP or HABP after treament with hyaluronidase (Fig. S8). This predominantly removes HA, but also chondroitin sulfate although with limited ability and slower kinetics^37^. The same 50% reduction in staining was observed with both MIP and HABP. Since HABP is specific for HA and does not bind to chondroitin sulfate, we can conclude that the observed staining by the MIP is predominantly that of HA.

Previously, rhodamine-based glucuronic acid-MIPs for bioimaging hyaluronic acid were synthesized by precipitation polymerization in an organic solvent, DMSO^34^. Due to their larger size (400 nm), these MIPs could target only extracellular hyaluronic acid. Additionally, they had a lower affinity and did not disperse well in aqueous medium, resulting in particle agglomeration and less efficient staining.

## Conclusion

The solid-phase synthesis method for preparing nanoMIPs was first introduced in 2013. It allows the generation of template-free, water-soluble, nanosized MIPs with homogeneous binding sites and high affinity, comparable to those of monoclonal antibodies. This method is based on the covalent immobilization of the template onto the surface of GBs, used as solid support. Only a few linking chemistries for immobilizing the template have been described so far, somehow restricting the wider application of this method. We showed that Cu(I)-catalyzed click chemistry can constitute an interesting alternative to immobilize a template on GBs. An azide-functionalized template, azidopropyl glucuronic acid was immobilized on GBs *via* their functionalized terminal alkyne groups. Besides, this convenient strategy allows the functional groups on the template, involved in molecular recognition, to be left free for imprinting. Similarly, azide-immobilized glass beads can be prepared if the template is preferentially available with an alkyne moiety. Due to the incorporation of a polymerizable benzamidine-based monomer enabling strong electrostatic interactions with the template’s –COOH group in the polymerization mixture, MIP-NPs with a K_D_ of ∼800 nM for the template were obtained. Endowed with a high specificity, small size and water-solubility, the MIP-NPs proved ideal to sense extracellular and intracellular HA. The procedure with MIP-NPs is fast, straightforward and comprises a versatile method for glycan bioimaging. Oriented solid-phase synthesis enables the obtention of a novel generation of MIPs that can efficiently compete with biological receptors. Thus MIP-NPs are promising diagnostic tools, and this methodology could be extended to the production of MIPs for other relevant cell biomarkers.

## Methods

### Reagents and instruments

These are described in Supplementary information.

### Synthesis of azidopropyl glucuronic acid

The synthesis and NMR characterization of azidopropyl glucuronic acid **4** (Figs S3 and S4) are reported in Supplementary Information.

### Immobilization of azidopropyl glucuronic acid on propargylated GBs

#### Preparation of propargylated GBs

GBs were first activated by boiling in 4 M NaOH (100 g of GBs in 100 mL of NaOH), for 10 min. The activated GBs were then washed with water and acetone and dried in an oven at 50 °C. Alkyne functionalization was done on 25 g activated GBs by incubation in 50 mL O-(propargyloxy)-N-(triethoxysilylpropyl)urethane 5% (v/v) in dimethyl formamide (DMF), in an oil-bath at 90 °C for 17 h, as previously reported^29^, with some modifications. The alkyne GBs were washed with DMF and acetone, dried in an oven at 50 °C and kept at 4 °C until further use.

#### Quantification of alkyne groups on GBs

The alkyne groups coupled to the GBs were quantified by using a fluorescent dye. Coumarin azide was clicked to the alkyne GBs via a copper (I) catalyzed Huisgen 1,3-dipolar cycloaddition of an azide and an alkyne (CuAAC) reaction^30^. Prior to the quantification, a calibration curve was generated with standard solutions of coumarin azide (0.25–7.5 µM) in a mixture of dimethyl sulfoxide (DMSO):water (1:1). The fluorescence was measured at 477 nm, with an excitation wavelength of 437 nm. For the quantification, different amounts (20–60 mg) of alkyne-GBs were incubated with 30 µL of a 1 mM stock solution of coumarin azide (30 nmol) in DMSO:water (1:1), 20 µL of 10 mM CuSO_4_∙5H_2_O (200 nmol) in water and 200 µL of 10 mM sodium ascorbate (2 µmol) in water, in a final volume of 1 mL DMSO:water (1 :1). After 6 h incubation at room temperature, the unbound coumarin azide was determined on the supernatant (3–10 fold dilution in DMSO:water (1:1) so as to fall within the calibration curve (Fig. S5). The amount of dye bound to the GBs was calculated by subtracting the amount of unbound dye from the amount obtained with the corresponding amount of activated glass beads without any functionalization (blank).

#### Immobilization of azidopropyl glucuronic acid

Immobilization of the template was performed via a CuAAC reaction. In a glass petri dish, 20 g of alkyne-GBs were incubated with 50 mg (0.18 mmol) azidopropyl glucuronic acid, 200 µL of 10 mM CuSO_4_∙5H_2_O (2 µmol) and 2 mL of 10 mM sodium ascorbate (20 µmol) in 10 mL water. The mixture was stirred in the dark for 17 h at room temperature. The glucuronic acid derivatized GBs (GlcA-GBs) were washed with water and dried in an oven at 37 °C.

### Solid-phase synthesis of MIP-NPs for glucuronic acid

Prior to MIP synthesis, the Lower Critical Solution Temperature (LCST) of the polymers was determined and found to be ~32 °C (Fig. S6). The solid-phase synthesis of MIP-NPs was carried out in a glass column equipped with a thermostated jacket (XK 26/40, GE Healthcare, Fontenay sous Bois, France), connected to a thermostated circulation water bath (Bioblock Scientific polystat 5, Fisher Scientific, France). The solvents were pumped through the column using a peristaltic pump at a flow rate of 2.5 mL/min. The column was packed with 20 g GlcA-GBs and washed with 100 mL of buffer A. Then 20 mL of an AB solution in buffer A (10 mg, 0.04 mmol, 5 mol %) was percolated through the column. The flow-through was collected and passed through the column again for 1 h to favor the AB-glucuronic acid interaction. The polymerization mixture was prepared by mixing the rest of the monomers, NIPAM (80 mg, 0.706 mmol, 85 mol%), EbAm (13.9 mg, 0.082 mmol, 10 mol%) and a polymerizable fluorescent rhodamine B dye (1.36 mg, 0.002 mmol) in a molar ratio of 0.05:1 (rhodamine B: AB), in 20 mL buffer A so that the total monomer concentration is 0.5% (v/v). The solution was purged with nitrogen for 30 min. Afterward, the initiation couple composed of KPS (7.34 mg in 500 µL buffer A) and TEMED (56 µL of 10 µL TEMED in 990 µL buffer A) was added to the reaction mixture. The latter was percolated through the reactor, and the temperature was set at 37 °C for 17 h (overnight). The column was then washed with 100 mL (5 × 20 mL) of buffer A at 37 °C, and the MIP-NPs were eluted with 3–5 fractions of 5 mL buffer A at room temperature (~25 °C). Control non-imprinted polymers (NIP) were synthesized with 20 g alkyne GBs, in the absence of glucuronic acid.

DLS measurements were performed on all eluted fractions, and MIPs which had similar sizes and dispersities (the first three fractions, i.e. 15 mL) were pooled. To determine the amount of MIP-NPs produced, 4 mL from the pooled fractions was centrifuged at 5,000 *g* using an Amicon Ultra-4 centrifugal filter unit with a MWCO of 100 kD (Merck-Millipore, France), for 1 h at 40 °C so as to remove buffer A and precipitate the polymers. The retentate (pink solid) was suspended in 1 mL water and lyophilized. The dry MIP-NPs were weighed on a precison balance, which allows to determine the concentration of the MIP-NPs (mg/mL) and the yield of polymerization, calculated as milligrams of MIP-NPs per gram of GBs.

### MIP-NPs characterization

#### Size determination

The hydrodynamic size of the MIP-NPs was measured directly on the eluate fractions from the column by DLS at 25 °C. MIPs having similar sizes and dispersities were pooled together and their size was determined by DLS analysis.

#### Fluorescence measurements

The pooled fraction was concentrated by centrifugation at 40 °C, 5,000 *g* using an Amicon-Ultra filter 100 K. The retentate was diluted with water to reach a final concentration of 2 mg/mL. Fluorescence measurements were done using an excitation at 540 nm, and emission wavelengths set at 548–650 nm, slit 3 nm using 1 mL MIP and NIP solution (2 mg/mL).

#### Evaluation of the binding properties

The binding properties of the polymers towards GlcA in water were evaluated by equilibrium binding experiments. A stock concentration of 2 mg polymer/mL in water was employed. Different concentrations of polymer particles (0.15–1.5 mg/mL) were pipetted in separate 2-mL polypropylene microcentrifuge tubes. After addition of radiolabeled glucuronic acid (100 pmol, 5 nCi), the final volume was adjusted to 1 mL with water and the mixture was incubated overnight at 37 °C on a tube rotator. The samples were centrifuged at 30,130 *g* for 1 h at 40 °C and a 500 µL aliquot of the supernatant was pipetted into a scintillation vial containing 4 mL of scintillation liquid. The amount of free radioligand was measured with a liquid scintillation counter and the amount of radiolabeled analyte bound to the polymer particles was calculated by subtracting the amount of the unbound analyte from the total amount of the analyte added to the mixture, determined from zero-polymer blanks.

### Cell culture

Human adult low calcium high temperature (HaCaT) cells were cultured in Dulbecco’s Modified Eagle Medium (DMEM)-high glucose with 10% fetal bovine serum (FBS) and 1% penicillin/streptomycin medium, hereafter referred as cell culture medium, at 37 °C, 5% CO_2_ and 100% humidity. Cells were passaged when confluent using 0.25% trypsin/ethylenediaminetetraacetic acid (EDTA) in PBS. For microscopic studies, the cells were cultured in 12-well plates (well diameter 22.1 mm) equipped with round glass cover slips (diameter 12 mm). 100 µL of 1 × 10^5^ suspended HaCaT cells (previously counted with a Malassez counting chamber from Marienfeld-Superior, Germany) were pipetted onto each cover slip. After 3 h of incubation, 2 mL medium was added to the wells and cells were left to grow to confluency for 48 h.

### Cell fixation and sample preparation for epifluorescence and confocal microscopy imaging

#### With MIP and NIP-NPs

Each cover slip with confluent HaCaT in 12-well plates was washed with 2 mL PBS and fixed at room temperature for 10 min in 600 µL paraformaldehyde (3% w/v) in PBS. To stop fixation, each cell sample was incubated 3 times with 1 mL 20 mM glycine in PBS for 20 min at room temperature and washed 3 times with 2 mL PBS. After fixation, the cells were washed 3 times with 1 mL water and incubated with 1 mL of 100 µg/mL polymer suspension in water at 37 °C for 90 min. Each fixed cell layer was washed 3 times with 1 mL water and the cell nucleus was stained by incubation with 600 µL of a Hoechst:PBS (1:1000) (v/v) solution at room temperature. After 10 min incubation, the samples were washed 3 times with 1 mL PBS and mounted for fluorescence microscopy imaging on a microscope slide with 5 µL mounting medium. The mounting medium consisted of 0.5 mL water, 0.5 mL 1 M Tris-HCl buffer pH 8 and 9 mL glycerol.

#### With HABP

HaCaT cells were grown on cover slips and fixed as described above. A stock solution of 50 µg HABP in 200 µL PBS containing 1% bovine serum albumin and 0.05% Tween 20 was prepared. 800 µL of 50 ng/mL HABP in PBS was incubated with the cell samples at 4 °C overnight. The cells were rinsed 3 times with PBS and incubated with 800 µL of 0.25 mg/mL streptavidin-FITC in PBS at 4 °C for 30 min, followed by 3 times rinsing with PBS. The cell nucleus was stained with Hoechst as mentioned above and the samples were mounted on a glass microscope slide with 5 µL mounting media.

#### Hyaluronidase-treated samples

HaCaT cells were grown on cover slips as described above. After fixation, cells were incubated for 90 min with 600 µL hyalyronidase (75 U) solution in PBS at 37 °C. The cells were washed 3 times with PBS and stained with MIP-NPs or HABP/streptavidin-FITC as described above.

Epifluorescence images were captured with a Leica DMI 6000B microscope, filter sets A4, L5 and TX2, N PLAN L 20.0 × 0.40 DRY, HCX PL FLUOTAR 40.0 × 0.60 DRY, HXC FLUOTAR 63.0 × 0.70 DRY and HCX FLUOTAR 100.0 × 1.30 OIL objectives with 20×, 40×, 63× and 100× magnification using exactly the same settings concerning light intensity and exposure time in 16-Bit Tiff format for each image. From each sample, at least 4 images were captured with the Leica Application Suite (LAS) software and each cell sample was at least prepared in double. All fluorescence intensities were determined with ImageJ (National Institute of Health, USA, version 1.46r). 3–4 images per sample with the same magnification were analyzed, by measuring the relative fluorescence of 10 cells per image after applying background substraction (area with no cells). Statistical significance for two groups’ comparison was calculated with Student’s t-test. Confocal microscopy images were captured with a Zeiss LSM 710, AxioObserver. A Plan-Apochromat 63×/1.40 Oil DIC M27 objective and 405 nm, 488 nm and 543 lasers were used for all images.

## Supplementary information

Supplementary information
