# Supplementary material for: Solid-phase synthesis of molecularly imprinted polymer nanolabels: Affinity tools for cellular bioimaging of glycans
Source: Sci Rep. 2019 Mar 8;9:3923. doi: 10.1038/s41598-019-40348-5 (PMC6408489; doi:10.1038/s41598-019-40348-5)
Supplement: Supplementary file 1 — Supplementary information [file 41598_2019_40348_MOESM1_ESM.pdf]

Supplementary information

Solid-phase synthesis of molecularly imprinted polymer nanolabels: Affinity tools for cellular bioimaging of glycans

Paulina X. Medina Rangel[1], Sylvain Laclef[2], Jingjing Xu[1], Maria Panagiotopoulou[1], José Kovensky[2,*]
Bernadette Tse Sum Bui[1,*] and Karsten Haupt[1,*]

[1]*Sorbonne Universités, Université de Technologie de Compiègne, CNRS Enzyme and Cell Engineering Laboratory, Rue Roger Couttolenc, CS 60319, 60203 Compiègne Cedex, France*

[2]*Université de Picardie Jules Verne, Laboratoire de Glycochimie, des Antimicrobiens et des Agroressources, UMR CNRS 7378, 33 rue Saint Leu, 80039 cedex Amiens, France*

Corresponding authors: karsten.haupt@utc.fr (Karsten Haupt), Fax: 33 344203910, Tel: 33 344234455; jeanne.tse-sum-bui@utc.fr (Bernadette Tse Sum Bui) and jose.kovensky@u-picardie.fr (José Kovensky)

Materials and Instruments

All chemicals and solvents were of analytical grade and purchased from VWR International (Fontenay sous Bois, France) or Sigma-Aldrich (St Quentin Fallavier, France), unless otherwise stated. Buffers were prepared with Milli-Q water, purified using a Milli-Q system (Millipore, Molsheim, France). (O-(propargyloxy)-N-(triethoxysilylpropyl)urethane was purchased from Abcr GmbH, Germany and coumarin 343 azide was from Interchim, France. Methacryloxyethyl thiocarbamoyl rhodamine B was purchased from Polysciences Europe GmbH, Germany. Biotinylated hyaluronic Acid Binding Protein (HABP) from Bovine Nasal Cartilage was purchased from Merck Millipore (France) and streptavidin-FITC was obtained from Sigma-Aldrich. Hyaluronidase type II from sheep testes was from Sigma-Aldrich. Glycine and paraformaldehyde (PFA) were from Applichem. HaCaT cells were obtained from Cell Lines Service (Eppelheim, Germany). Glass cover slips, cell culture flasks (crystal-grade polystyrene), 12 wellplates (crystal-grade polystyrene), penicillin/streptomycin, Hoechst 33342 trihydrochloride trihydrate 10 mg/mL solution in water, Dulbecco's Modified Eagle Medium (DMEM), fetal bovine serum (FBS), 0,25% trypsin/EDTA and phosphate-buffered saline pH 7.4 (PBS) were from Thermo Fisher Scientific (Illkirch, France). Glass beads of diameter 0.1 mm and microscope slides for cell samples were from Roth Sochiel E.U.R.L. (Lauterbourg, France). D-[6-^{14}C]glucuronic acid (specific activity: 55 mCi/mmol, activity: 0.1 mCi/mL) was from Biotrend Chemikalien GmbH (Köln, Germany). Radioactivity was measured in the presence of scintillation liquid (Ultra Gold, PerkinElmer, France) with a liquid scintillation counter (Beckman LS-6000 IC). Dynamic light scattering (DLS) analysis was performed on a Zeta-sizer NanoZS (Malvern Instruments Ltd., Worcestershire, UK). Fluorescence measurements were done on a FluoroLog-3 spectrofluorimeter (Horiba Jobin Yvon, Longjumeau, France).

Synthesis of azidopropyl glucuronic acid

Methods for compound purification and characterization. All reagents were used without further purification. Dichloromethane was distilled over calcium hydride. Analytical thin-layer chromatography (TLC) was carried out on DC Kieselgel 60 F254 Alufolien (Merck-Millipore). Flash chromatography was performed on a Reveleris iES System using silica cartridges and an evaporative light scattering detector (ELSD)/UV detection (Grace, USA). ^{1}H and ^{13}C nuclear magnetic resonance (NMR) spectra were respectively recorded at 400 and 100 MHz using a Bruker DRX-400 spectrometer. Chemical shifts are reported in parts per million relative to a reference. For D_2O, isopropyl alcohol was used as standard for ^{13}C (^{1}H: δ = 1.17 ppm, ^{13}C: δ = 24.38 ppm). Peak multiplicity is reported as doublet (d), triplet (t) or multiplet (m). High-resolution mass spectra (HRMS) were obtained by electrospray ionization using a Micromass-Waters Q-TOF Ultima Global instrument.

Chemical synthesis. 1-O-(3-azidopropyl)-D-glucopyranuronic acid **4** (Fig. 1), which we term azidopropyl glucuronic acid in the text, was synthesized following a reported procedure[1]. The original protocol was modified in order to obtain compound **4** without the purification of intermediates. Compound **4** was

obtained as a 2.8:1 mixture of anomers in 35 % overall yield. More in detail, methyl 1,2,3,4-tetra-*O*-acetyl-D-glucopyranuronate **2** was first obtained from commercially available D-glucurono-6,3-lactone **1** as described[1]. To obtain methyl 2,3,4-tri-O-acetyl-1-O-(3-chloropropyl)-D-glucopyranuronate **3**, compound **2** (5 g, 13.29 mmol) was dissolved in dry CH_2Cl_2 (125 mL) under argon, followed by the addition of 3-chloropropanol (3.4 mL, 39.86 mmol, 3 equiv.). The mixture was stirred for 30 min at room temperature under argon. After letting it to cool to 0 °C, $BF_3{\cdot}O(C_2H_5)_2$ (1.7 mL, 13.29 mmol, 1 equiv.) was added dropwise. The reaction was stirred at room temperature for 12 h. After completion of the reaction indicated by TLC, the mixture was filtered and the filtrate was washed with saturated $NaHCO_3$. The organic layer was evaporated under reduced pressure yielding a mixture of anomers which was used in the next step without further purification.

1-O-(3-azidopropyl)-D-glucopyranuronic acid **4** was obtained as follows. The precedent mixture was dissolved in dimethylformamide (DMF) (125 mL) and sodium azide (4.31 g, 66.45 mmol, 5 equiv.) was added. The reaction mixture was stirred at 80 °C for overnight. The solvent was rotary evaporated. The resulting mixture was dissolved in methanol (125 mL) and K_2CO_3 (184 mg, 1.33 mmol) was added. The suspension was stirred at 25 °C for overnight. Water (125 mL) and potassium hydroxide (2.24 g, 40 mmol, 3 equiv.) were added. After being stirred at room temperature for 4 h, the reaction mixture was neutralized with DOWEX HCR-W2 (H^+) resin, filtered and concentrated. The residue was purified by silica gel chromatography (6:2:1 EtOAc/MeOH/H_2O) to afford a pure colorless oil as a 2.8:1 mixture of 1-*O*-(3-azidopropyl)-β-D-glucopyranuronic acid and 1-*O*-(3-azidopropyl)-α-D-glucopyranuronic acid in 35% overall yield. Major anomer β: NMR (400 MHz, 25 °C, D_2O): δ_H 4.53 (d, J = 8.0 Hz, 1H), 3.99-4.05 (m, 1H), 3.90 (d, J = 9.4 Hz, 1H), 3.76-3.82 (m, 1H), 3.67-3.60 (m, 1H), 3.55-3.59 (m, 2H), 3.49 (t, J = 6.6 Hz, 2H), 3.33-3.37 (m, 1H), 1.91-2.00 (m, 2H). ^{13}C NMR (100 MHz, 25 °C, D_2O): δ_C 174.02, 102.42, 75.53, 75.37, 72.98, 71.67, 67.65, 48.02, 28.39. NMR characterization of the major product (Figs. S3 and S4) corresponds to 1-*O*-(3-azidopropyl)-β-D-glucopyranuronic as described[1]. HRMS (ESI^-): Calculated for $C_9H_{15}N_3O_7$ $[M-H]^-$: m/z 276.0837. Found: 276.0843.

Synthesis of 4-acrylamidophenyl)(amino)methaniminium acetate

The functional monomer 4-acrylamidophenyl)(amino)methaniminium acetate (AB) was synthesized as previously reported[2]. Briefly, 4-acrylamidophenyl)(amino)methaniminium chloride was first synthesized. For this, 34 g (0.25 mol) of sodium acetate trihydrate was dissolved in 200 mL of water and 2 g (9.6 mmole) of 4-aminobenzamidine dihydrochloride was added. The solution was cooled to < 5 °C in an ice bath and 4 mL (49 mmol) of acryloyl chloride was added dropwise. The reaction was left to proceed for 1 h. The pH was then adjusted to 4.0 with hydrochloric acid (37 %) and precipitation was observed. After filtration, the precipitate was redissolved in 100 mL of water at 40 °C. Hydrochloric acid was again added this time to pH 1.0 and the product was left overnight to crystallize at 4 ºC. The crystals were collected by filtration and dried in an oven maintained at 50 °C. The yield of 4-acrylamidophenyl)(amino)methaniminium chloride

was 60%. ^{1}H NMR (400 MHz, DMSO-d_6): 10.56 (s, 1H), 8.99 (s, 4H), 7.84 (d, 2H), 7.81 (d, 2H), 6.48 (d, 1H), 6.31 (dd, 1H), 5.82 (s, 1H).

4-arylamidophenyl)(amino)methaniminium chloride was then converted to 4-acrylamidophenyl)(amino)methaniminium acetate as the acetate ion is more readily exchangeable with the template's carboxylate. Therefore 1.0 g of 4-acrylamidophenyl)(amino)methaniminium chloride was suspended in 100 mL of saturated sodium acetate solution and stirred overnight. The product was collected by filtration, washed with water to eliminate residual sodium acetate and dried at 50 °C. The yield of 4-acrylamidophenyl)(amino)methaniminium acetate, which we term AB in the text, was 60 %. ^{1}H NMR (400 MHz, DMSO-d_6):10.56 (broad s, 5H), 7.84 (d, 2H), 7.78 (d, 2H), 6.48 (dd, 1H), 6.31 (dd, 1H), 5.82 (dd, 1H), 1.70 (s, 3H).

Determination of Lower Critical Solution Temperature (LCST) of the polymers

Prior to MIP synthesis, the polymerization mixture was prepared in a glass vial by mixing the functional monomers NIPAM (40 mg, 85 mol %) and AB (5 mg, 5 mol %), together with the crosslinker *N,N'*-ethylenebis(acrylamide) (EbAm) (6.9 mg, 10 mol %) in a total volume of 10.3 mL of 25 mM sodium phosphate buffer, pH 7.0 (buffer A). The total monomer concentration is 0.5% (w/w). Then, 3.6 mg potassium persulfate (KPS) and 28 µL of *N,N,N',N'*-tetramethylethylenediamine (TEMED) from a stock solution of (10 µL in 990 µL buffer A) (7.5/1 molar ratio, the amount of KPS was 3% mol/mol with respect to polymerizable double bonds), as initiation system was added. The mixture was purged with nitrogen for 30 min and left to polymerize overnight in a water-bath at 37 °C. The LCST was determined in a 1-cm path glass cuvette by scanning the temperature of the polymer and monitoring its size change by dynamic light scattering. The polymer mixture was heated automatically from 22 to 54 °C (1 °C/min), with a delay time of 2 min between each temperature before taking the measurements. The LCST was found to be ~32 °C (Fig. S6).

Molecular weight determination

The molecular weight of MIP in buffer A was obtained by analysis of the intensity of light scattered from the particle at different concentrations, in the dilute regime. This is done by applying the Rayleigh equation[3]:

$KC/R_\theta = 1/M + 2A_2C$

R_θ is the Rayleigh ratio, the ratio of scattering of the sample compared to the scattering of a standard (toluene), K is an optical constant, C is the sample concentration, M is the molecular weight and A_2 is the second virial coefficient (representing the magnitude of particle - solvent interactions). To determine the molecular weight, the Rayleigh equation is represented graphically in the form of a Debye plot showing the concentration (C) dependence of intensity of scattered light (KC/R_θ). M is determined from the intercept on the y-axis and A_2 from the slope of the Debye plot.

Concentrations of MIPs varying between 0.2 to 0.8 mg/mL in water were used for measurements. The molecular weight was determined on a Zetasizer NanoZS at 25 °C. The Debye plot (y = 0.0005x + 0.0016, R^2 = 0.8363) is shown in Fig. S7, which indicates a molecular weight of 625 ± 35 kDa and a second virial coefficient of 0.0005 mL mol/g^2. A positive A_2 indicates that the polymer-solvent interaction is stronger than the polymer-polymer interaction; therefore the MIP will tend to stay as a stable solution.

Figures

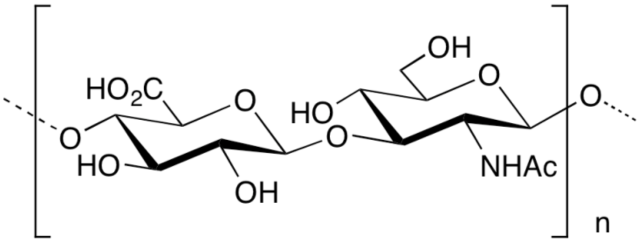

Fig. S1. Chemical structure of hyaluronic acid, repeated disaccharide units of D-glucuronic acid and N-acetyl-D-glucosamine.

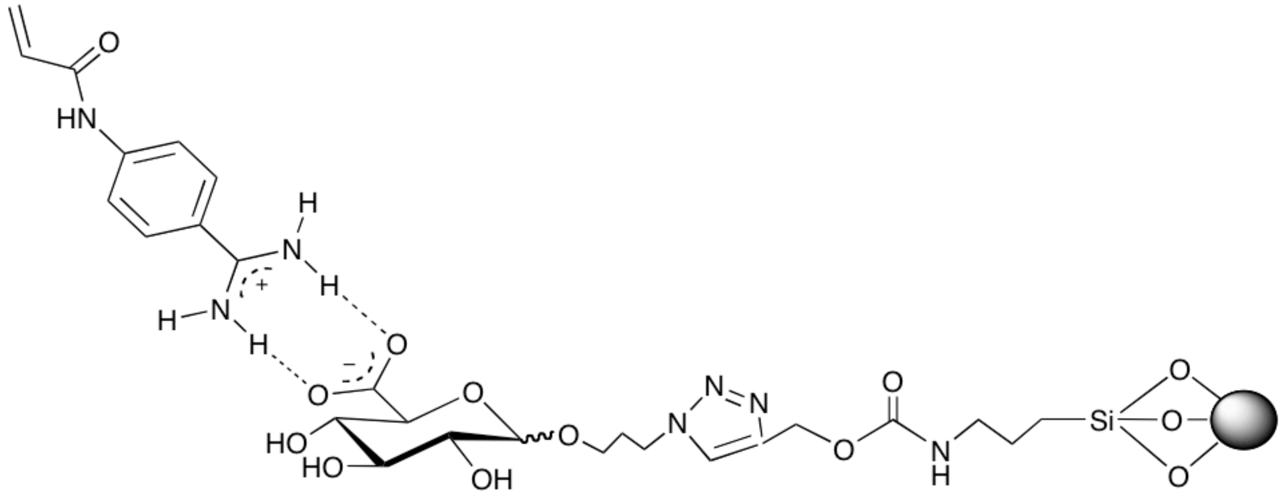

Fig. S2. Oriented immobilization of the template. Azidopropyl glucuronic acid is immobilized by click reaction on propargylated GBs, generating a 1,2,3-triazole group. AB monomer added in the polymerization mixture forms strong electrostatic interactions with the –COOH moiety of glucuronic acid.

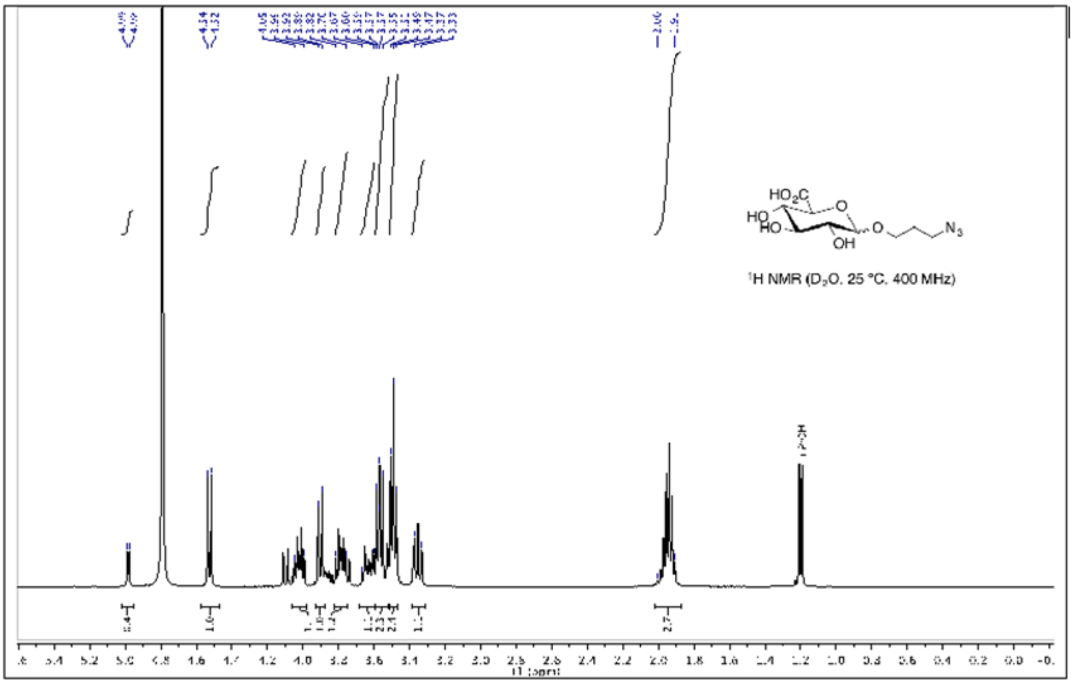

Fig. S3. ^{1}H NMR spectrum of azidopropyl glucuronic acid in D_2O.

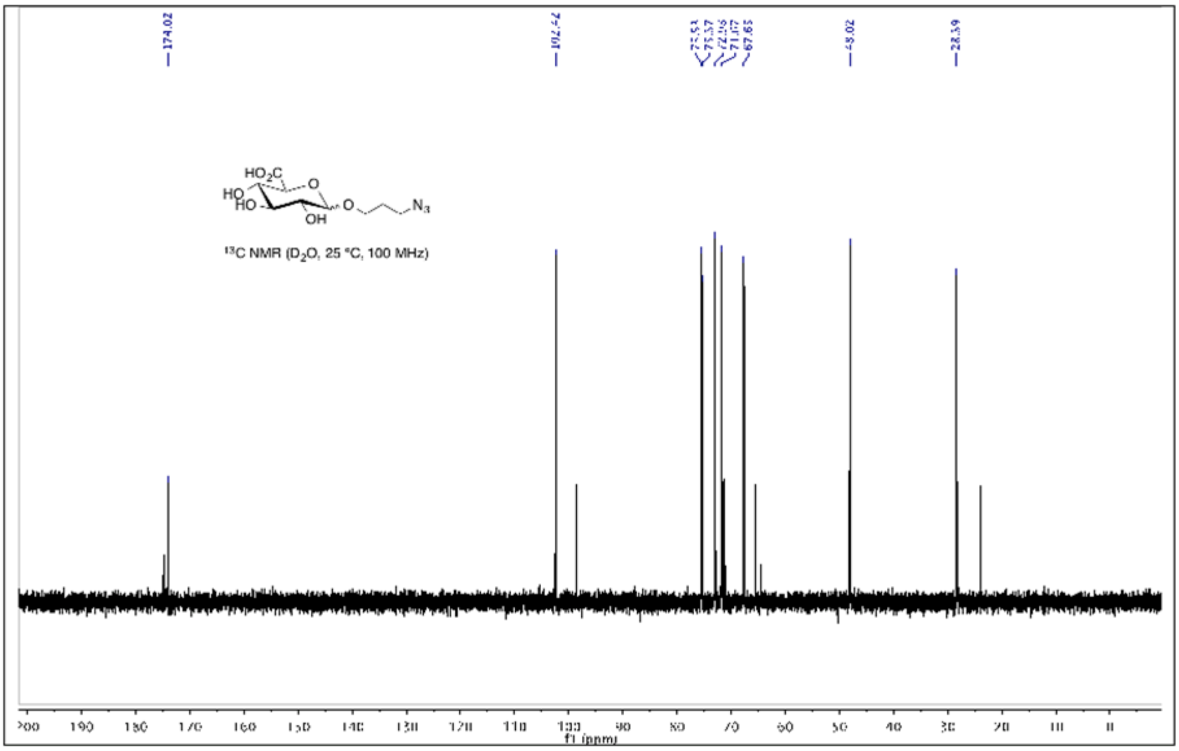

Fig. S4. ^{13}C NMR spectrum of azidopropyl glucuronic acid in D_2O.

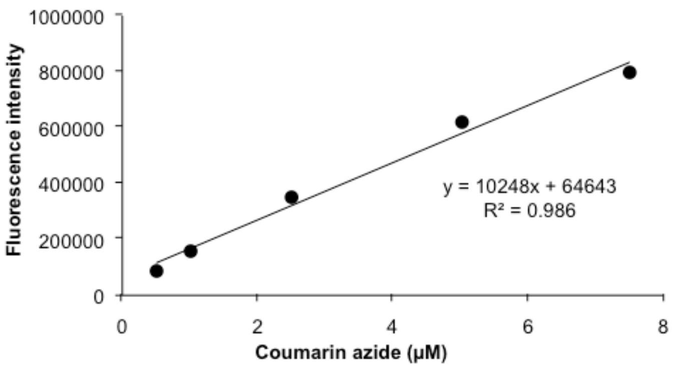

Fig. S5. Representative calibration curve of coumarin azide in DMSO:water (1:1). λ_{ex} = 437 nm and λ_{em} = 477 nm, slit 1 nm.

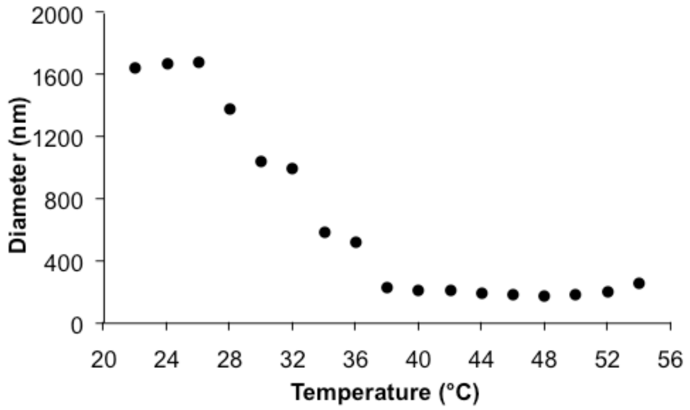

Fig. S6. Temperature scan of NIP-NPs in 25 mM sodium phosphate buffer, pH 7.0 (n = 2).

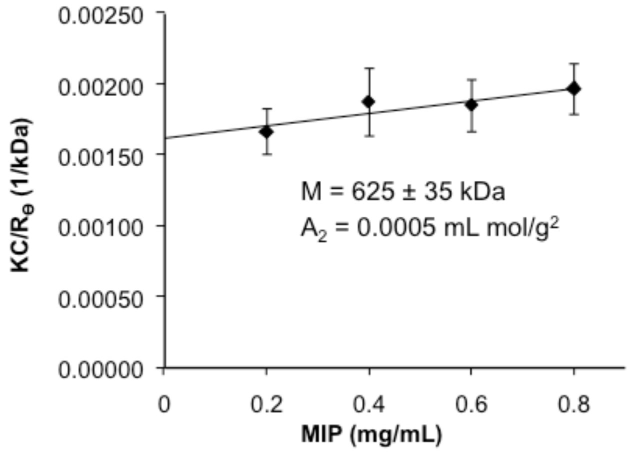

Fig. S7. Debye plot for the determination of the absolute molecular weight of MIP (n = 4).

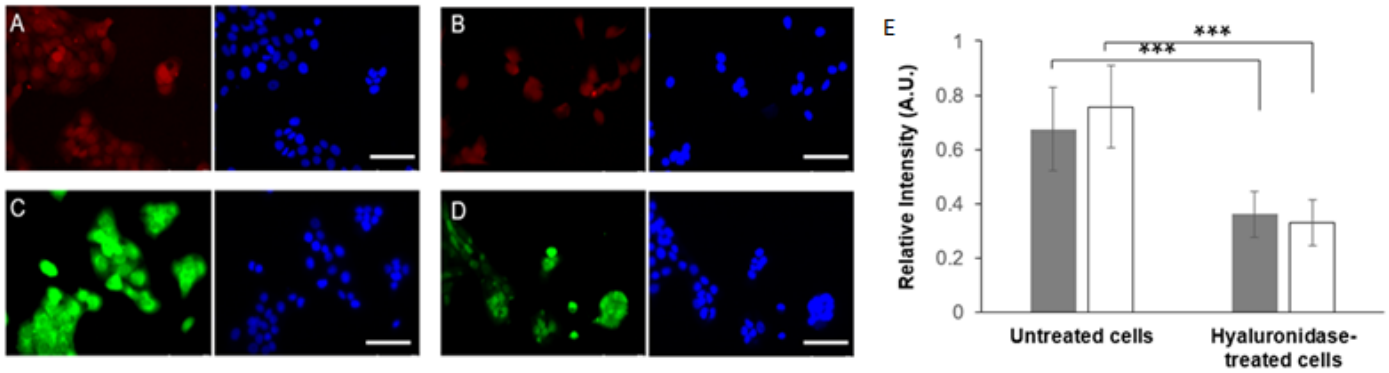

Fig. S8. (A-D) Epifluorescence images of HaCaT cells that were fixed ans stained with MIPs (red) (A,B) or HABP/streptavidin-FITC (green) (C,D) in untreated (A,C) and hyaluronidase-treated samples (B,D) with their corresponding cell nucleus counterstained with Hoeschst (blue). Scale bar: 75 µm. (E) Relative intensity of HaCaT cells treated or not with hyaluronidase prior MIP (grey) and HABP (white) staining (n=3 independent replicates with triplicates for each experiment). 4 images per sample with the same magnification were analyzed, by measuring the relative fluorescence of 10 cells per image after applying background substraction (area with no cells). Statistical significance for two groups' comparison was calculated with Student's t-test. Mean values of untreated and hyaluronidase-treated cells are significantly different at 95 % confidence ($p < 0.001$***, Student's t-test with equal variance).

References

1. Chen, Y., Li, Y., Yu, H., Sugiarto, G., Thon, V., Hwang, J., Ding, L., Hie, L., Chen, X. *Angew. Chem. Int. Ed.* **52**, 11852–11856 (2013).

2. Nestora, S., Merlier, F., Beyazit, S., Prost, E., Duma, L., Baril, B., Greaves, A., Haupt, K., Tse Sum Bui, B. *Angew. Chem. Int. Ed.* **55**, 6252–6256 (2016).

3. George, A., Wilson, W.W. *Acta Crystallogr. D Biol. Crystallogr.* **50**, 361–365 (1994).
